# A Novel Quantitative Arousal-Associated EEG-Metric to Predict Severity of Respiratory Distress in Obstructive Sleep Apnea Patients

**DOI:** 10.3389/fphys.2022.885270

**Published:** 2022-06-22

**Authors:** Malatantis-Ewert S, Bahr K, Ding H, Katharina Ludwig, Koirala N, Huppertz T, Gouveris H, Muthuraman M

**Affiliations:** ^1^ Department of Otorhinolaryngology, Sleep Medicine Center, Medical Center of the University of Mainz, Mainz, Germany; ^2^ Movement Disorders and Neurostimulation, Department of Neurology, Biomedical Statistics and Multimodal Signal Processing Unit, Medical Center of the University of Mainz, Mainz, Germany; ^3^ Haskins Laboratories, Yale University, New Haven, CT, United States

**Keywords:** sleep apnea, polysomnography, arousal index, area under the curve of arousal, support vector regressor, hypoxic burden, AHI, ODI

## Abstract

Respiratory arousals (RA) on polysomnography (PSG) are an important predictor of obstructive sleep apnea (OSA) disease severity. Additionally, recent reports suggest that more global indices of desaturation such as the hypoxic burden, namely the area under the curve (AUC) of the oxygen saturation (SaO2) PSG trace may better depict the desaturation burden in OSA. Here we investigated possible associations between a new metric, namely the AUC of the respiratory arousal electroencephalographic (EEG) recording, and already established parameters as the apnea/hypopnea index (AHI), arousal index and hypoxic burden in patients with OSA. In this data-driven study, polysomnographic data from 102 patients with OSAS were assessed (32 female; 70 male; mean value of age: 52 years; mean value of Body-Mass-Index-BMI: 31 kg/m^2^). The marked arousals from the pooled EEG signal (C3 and C4) were smoothed and the AUC was estimated. We used a support vector regressor (SVR) analysis to predict AHI, arousal index and hypoxic burden as captured by the PSG. The SVR with the arousal-AUC metric could quite reliably predict the AHI with a high correlation coefficient (0,58 in the training set, 0,65 in the testing set and 0,64 overall), as well as the hypoxic burden (0,62 in the training set, 0,58 in the testing set and 0,59 overall) and the arousal index (0,58 in the training set, 0,67 in the testing set and 0,66 overall). This novel arousal-AUC metric may predict AHI, hypoxic burden and arousal index with a quite high correlation coefficient and therefore could be used as an additional quantitative surrogate marker in the description of obstructive sleep apnea disease severity.

## Introduction

In 2008 it has been stated that 3–7% of adult men and 2–5% of adult women in populations at risk for sleep disordered breathing or cardiovascular diseases have sleep apnea syndrome. There has been a 14–55% increase in prevalence of obstructive sleep apnea (OSA) over the last 20 years. In particular, patients with cardiovascular disease have been found to have a two-to threefold increased prevalence relative to the normal population (Young et al., 2002; Punjabi, 2008). In a large population-based study (“HypnoLaus study”) the prevalence of moderate-to-severe sleep-disordered breathing was even higher, with 23·4% in women and 49·7% in men (Heinzer et al., 2015).

The pathogenesis of sleep-disordered breathing is based on central nervous and/or neuromuscular processes that lead to changes in central respiratory regulation and/or upper airway muscle tone during sleep. However, the exact pathogenesis is still the focus of research and not completely understood.

Obstructive sleep apnea is diagnosed using polysomnography (PSG) or home sleep testing (HST). (Markun and Sampat, 2020). In this process, obstructive sleep apnea is diagnosed when the breathing disorder cannot be explained by any other sleep disorder, medical condition, medication, or other substance. In addition, to meet the diagnostic criteria, an apnea-hypopnea index (AHI) > 15/h (each event lasting ≥10 s) of sleep time or an AHI ≥5/h of sleep time in combination with typical clinical symptoms or relevant comorbidity must be present (Darien, 2014). The evaluation of PSG/HST adheres to the evaluation criteria of the American Association of Sleep Medicine (AASM). The main clinical findings are daytime sleepiness, including involuntary falling asleep, and the AHI, which objectifies the diagnosis and, in conjunction with the clinical symptoms, determines the severity of the disease. An AHI between 15/h and 30/h sleep time classifies OSA as moderate. In the range of an AHI >30/h sleep time, OSA is referred to as severe (Mayer et al., 2016).

However, in recent years, consensus is emerging within the sleep medicine community that the AHI metric may not be sufficient as a singular assessment parameter for classifying the severity of OSA. This metric has many limitations to stand as the sole parameter for defining severity. (Malhotra et al., 2021). Beginning with the fact that there are multiple definitions of hypopnea, the index of apnea and hypopnea provides no information about the length of each event or the severity of desaturation. Similarly, it is subject to the assumption that apneas and hypopneas should be evaluated equally in their disease-promoting effect (Punjabi, 2016; Randerath et al., 2018). Also, the AHI has a poor correlation with the clinical manifestation of OSA, such as daytime sleepiness, and does not have good predictive power about the risks for cardiovascular disease (CVD) resulting from the condition (Kulkas et al., 2013; Cao et al., 2020).

Therefore, the search for new parameters and novel metrics that provide a more precise prediction of adverse outcomes (cardiovascular, neurocognitive and metabolic, among others) continues. Polysomnography yields a valuable variety of data that should be used to describe the disease. New methods of measurement of respiratory variables and new technologies can better evaluate the different pathophysiological mechanisms underlying OSA (Randerath et al., 2018). One of the new and so far promising parameters in the PSG raw data is the so-called “hypoxic burden” (Cao et al., 2020). Hypoxic burden has been defined as the “total area under the respiratory event-related desaturation curve” (Azarbarzin et al., 2019). Hypoxic burden has been associated with increased CVD mortality in adults aged >40 years in two large cohort studies, namely the Outcomes of Sleep Disorders in Older Men (MrOS) and the Sleep Heart Health Study (SHHS). Higher blood pressure and risk of heart failure in men were also associated with hypoxic burden after eliminating some confounders, such as comorbidities (Azarbarzin et al., 2019; Azarbarzin et al., 2020).

Arousals can be spontaneously, physiologically and an integral part of healthy sleep regulation but also an indication of serious diseases, such as the sleep apnea syndrome we studied (Strollo and Rogers, 1996; Dvir et al., 2018). In 2007, the Arousal Task Force acknowledged in a systematic review that arousal has a major impact on the sleep process. Arousals are scored as an all-or-none event and defined as an abrupt shift of the EEG frequency including alpha, theta and/or frequencies greater than 16 Hz (but not spindles) that lasts at least 3 s, with at least 10 s of stable sleep preceding the change. Patients’ subjective and objective excessive daytime sleepiness (EDS), as one of the clinically leading symptoms, correlates positively with EEG arousal count. With increase in EEG arousal number, patients’ psychomotor performance also decreases, hormone secretions change, upper respiratory function decreases, sensory arousal threshold increases, and metabolic activity increases (Bonnet et al., 2007). Chemical factors as blood pressure, CO-2 partial pressure or oxygen saturation are believed to act as stimuli for triggering respiratory arousals (RAs) when reaching a certain threshold value (Younes, 2008). Studies showed that the maximal desaturations of SaO2 during respiratory events with arousals are larger than desaturations in events without arousals (Yan et al., 2016). However, it should be noted that arousal is not only associated with negative effects. The immediate physiological changes associated with arousal are beneficial in rapidly alleviating severe respiratory events and their sequelae (Eckert and Younes, 2014). But among patients the amount of stimuli that lead to an arousal seem to differ, as well as within the same individual for one night (Berry and Gleeson, 1997; Berry et al., 1998; Sforza et al., 1999). Conversely the number of stimuli that lead to an opening of the upper airway differ among individuals; but seem to be fixed regarding any given patient during sleep (Younes et al., 2007; Loewen et al., 2009). Not only the frequency (as depicted by the arousal index), but also the individual intensity of respiratory arousal is quite strong correlated to the OSA severity. Over all there is evidence that the microstructure of respiratory arousals may be patient-specific and that each OSA patient may have a cortical or sub-cortical neural arousal-associated pattern generator, which reacts to an obstructive respiratory event with a stimulus and a specific signature in terms of duration and intensity, like a distinct pattern, in order to ensure ventilation during sleep (Bahr et al., 2021). Overall, it is also not yet fully understood whether it is obstruction per se or the associated hypoxia that leads to arousals.

Overall, however, it can be stated, arousal is an important parameter in understanding the extent to which clinical symptoms are related to respiratory disturbances during sleep and the resulting treatment decisions. The primary aim of our present study was to identify possible associations between the AUC of respiratory arousal as a new metric index and already known parameters such as the hypoxic burden, AHI and arousal index in patients with OSA. Likewise, the arousal-AUC should provide another building block for a better understanding of the origin of arousal. Furthermore, we discuss the results in terms of suggestions for their further clinical use.

## Material and Methods

To correlate the area under the curve of the respiratory arousal with the hypoxic burden, AHI and arousal index in OSAS patients, PSG data were used from patients who underwent inpatient polysomnography for the initial diagnosis of obstructive sleep apnea, monitored by sleep medicine qualified personnel. Included in the recording, according to the international AASM standards, were a sleep electroencephalography (EEG), electrooculogram (EOG), electromyography (EMG), electrocardiography (ECG), respiratory flow, snoring, respiratory effort, oxygen saturation, body position, and a video recording during sleep. Nasal airflow was detected by measurement of impact pressure through a nasal sensor that determined pressure fluctuations of the breathed air stream. Thoracic and abdominal excursions, oxyhemoglobin saturation (pulse oximeter) and body position were simultaneously recorded. Snoring was recorded with a pre-laryngeally fixed microphone. The sampling frequency of the EEG data was 200 Hz. The data was high pass filtered at 0.1 Hz and was not resampled before the analyses. The two central channels (C3 and C4) were used for the analysis with the knowledge that many sleep laboratories around the world still use the Rechtschaffen & Kales EEG recordings (Kales and Rechtschaffen, 1968). The polysomnographic recordings were performed using the Alice-LE-Diagnostic Sleep System (Philips Healthcare/Respironics, Best, Netherlands as supplied by Loewenstein Medical, Bad Ems, Germany). In all patients, two PSG were performed on two consecutive days, and only the second night was used for the analysis of the data in each case to minimize any potential first-night effect on sleep efficiency and potentially minimize the possibility of missing a severe OSAS in the diagnosis (Gouveris et al., 2010). In the morning following each sleep study night, sleep stages and sleep-related respiratory events were manually scored according to the American Academy of Sleep Medicine (AASM)-2012 guidelines (Berry et al., 2012). This was performed visually by sleep medicine board-certified specialists. Nasal airflow amplitude reduction greater than 90%, lasting for at least 10 s, was defined as apnea. Hypopnea was defined as an airflow reduction between 50 and 90% with an associated 3% reduction of the blood oxygen saturation (SpO2). Apnea events were further classified into obstructive, central, or mixed based on simultaneous evaluation of nasal airflow and thoracic and abdominal excursion. Physiological EEG arousals (e.g., the one associated with changes in sleep stage) and motor-related arousals were excluded in this study.

Each patient also underwent a clinical examination prior to polysomnography, which adhered to the criteria of the DGSM (German Society for Sleep Research and sleep medicine) S3 guideline 2017 which is based on the guidelines of the AASM Manual for the scoring of sleep and associated events (Berry et al., 2012; Mayer et al., 2016).

Criteria for data inclusion and analysis, were a first diagnosis of OSAS with an AHI ≥15/h. Exclusion criteria were age <18 years, active malignant tumors (end of last therapy <5 years), COPD (Gold 2–4), Raynaud’s syndrome (due to problems with oxygen saturation measurement), congestive heart failure (NYHA III or IV), severe psychiatric illness, severe insomnia or pre-existing therapy for a known OSAS. Approval for the study was provided by the local Institutional Review Board (Nr. 2018–13942). The research findings presented in this manuscript are based on research and clinical practices that conform to the principles of the Helsinki Declaration.

**FIGURE 1:**
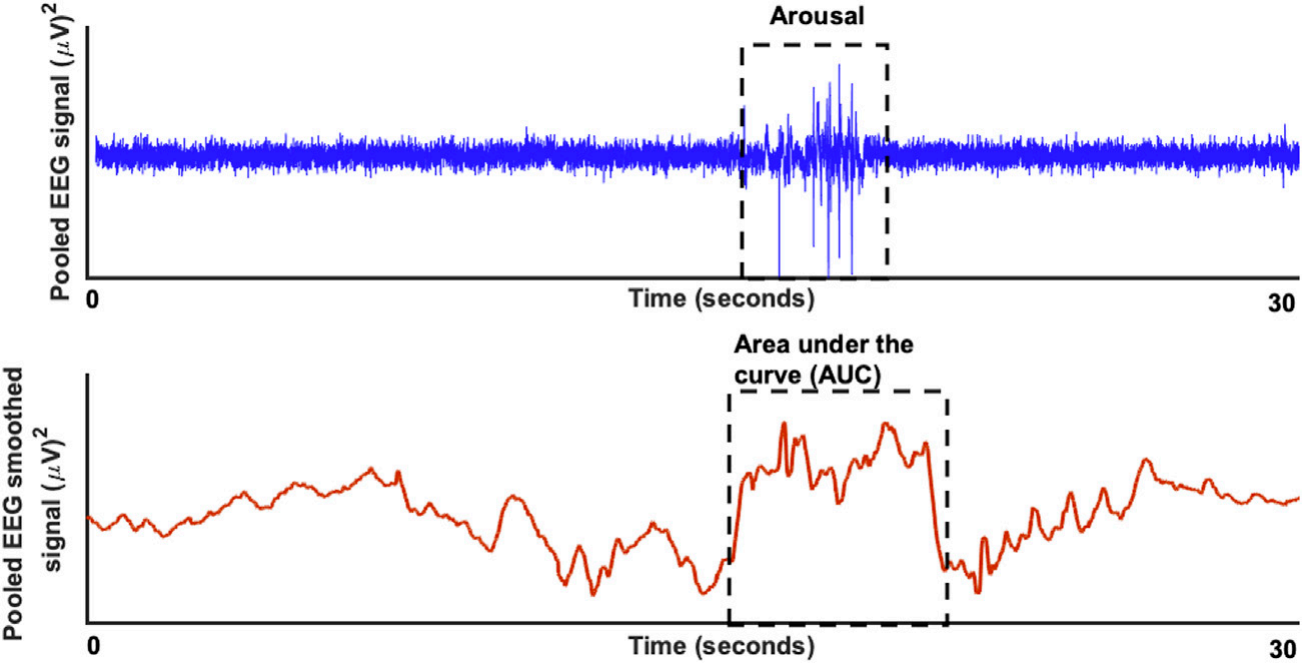
The raw EEG signal (in blue) for a duration of 30 seconds is shown in the top plot with a significant respiratory arousal. In the bottom blot (in red) the smoothed version of the EEG signal is shown to estimate the area under the curve.

**FIGURE 2:**
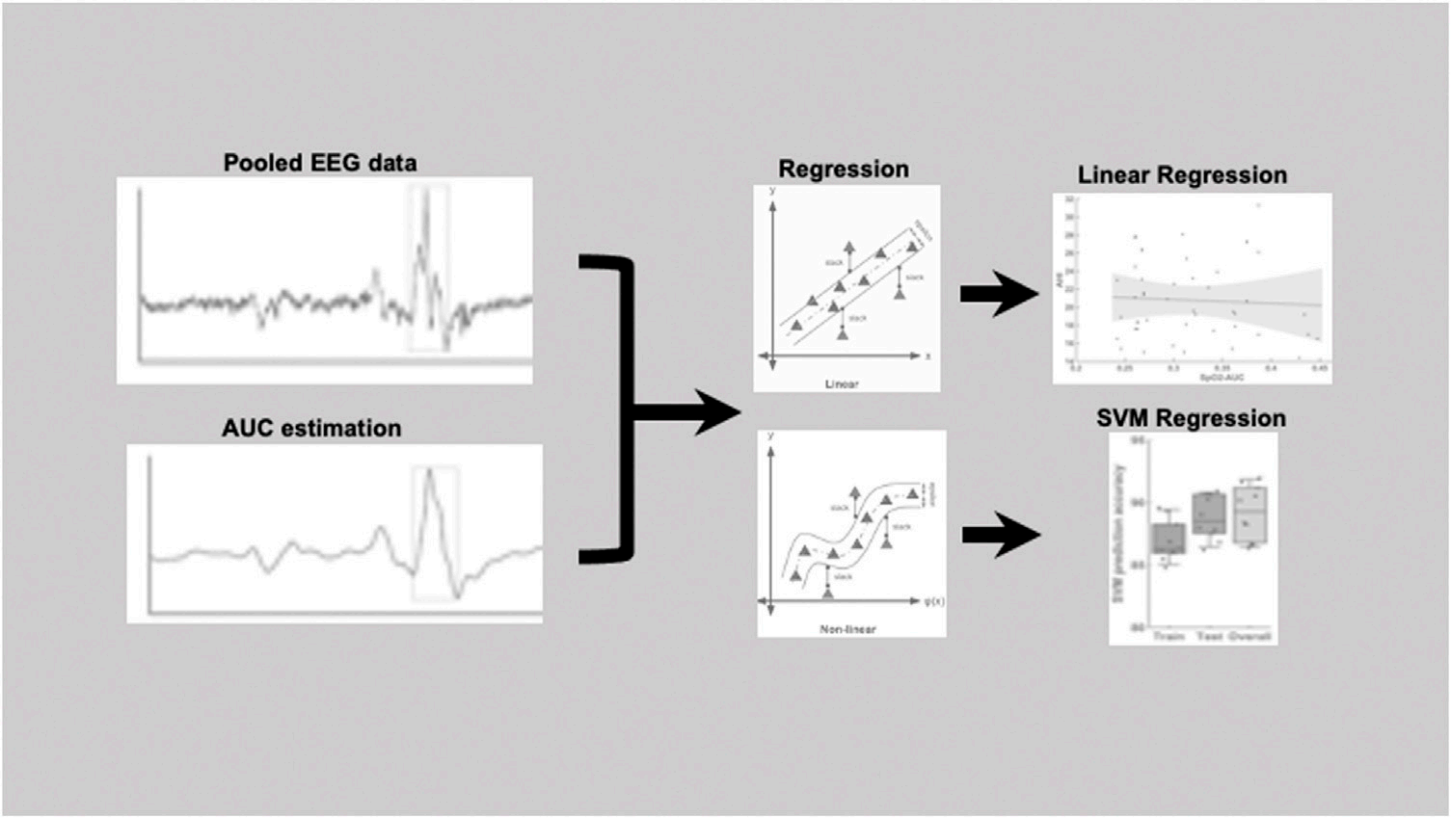
Pipeline figure to show the procedure from the raw EEG data, estimating the area under the curve (AUC) and then followed by the linear as well as the SVM regression.

Figure 1 shows an example of a pooled EEG trace in the top plot and the smoothed curve below from which the area under the curve was estimated. The pooling was done to increase the signal-to-noise ratio for the further analyses of area under the curve (AUC). In this study, the technique used was pooling together signals from multiple EEG channels (C3 and C4) weighted by their respective signal-to-noise (SNR) relative to the overall SNR of both the channels. The SNR was estimated in the raw signal, by taking „signal” component as the mean ±2 standard deviation and the „noise” component as the mean ±0,5 standard deviation. The values to indicate the SNR’s for each channel separately were: C3 (24,42 ± 4,84)dB, C4 (22,39 ± 5,51)dB and for the pooled signal SNR (31,23 ± 3,15)dB. The EEG signal was then smoothed based on the 200-time points average (sampling frequency of 200 Hz) equivalent to one second epochs. After the smoothing the area under the curve was determined based on the starting and end point was manually marked for each arousal. The slope was estimated from the starting point to the neighboring peak and followed by estimation of the AUC as illustrated in the schematic Figure 1. Here, we performed both a linear regression as well as a support vector regressor (SVR) analysis, representing a machine learning-based multiple regression method that could associate the observed and trained values and present the correlation coefficient as a prediction (Drucker et al., 1996). Figure 2 shows the procedure from the raw EEG data, estimating the area under the curve (AUC) and then followed by the linear as well as the SVM regression. To create a better comparison, we not only created a correlation between arousal-AUC and SpO2-AUC, but also between AHI and SpO2-AUC.

In this study, a data-driven regression model was implemented without explicitly stating a functional form indicating a nonparametric technique. In short, the algorithm looks for an optimally separating threshold between the two data sets by maximizing the margin between classes’ closest points. The points lying on the boundaries are called support vectors, and the middle of the margin is the optimal separating threshold. In most cases the linear separator is not ideal; therefore, a projection into a higher-dimensional space is performed where the data points effectively become linearly interrelated. Here, we have used the RBF kernel for this projection due to its good performance as discussed in Cortes and Vapnik (1995) and based on previous application of support vector machines in earlier studies (Cortes and Vapnik, 1995; Muthuraman et al., 2016; Michels et al., 2017; Michels et al., 2021). Then used the grid search (min = 1; max = 10) to find the few optimal input regularization parameters, namely C (Type of classification algorithm), which is the capacity constant. The parameter C should be carefully chosen because the larger the C, the more the error is penalized (i.e., leads to over-fitting) so we tested values in the range of 1–1,000 and choose a gamma of 0.25 for the RBF kernel function (which represents the data for the cross validation). The selection was checked by 10-fold cross-validation by taking 75% of the data set for training and 10% for testing. A soft-margin classifier of the calculated independent variables was used for every parameter and spurious correlations (correlations which could be due to artifacts) were weighted by a penalty constant P. To optimize correlation coefficient, this was calculated for every regressor. To demonstrate that no over-fitting is attested in our data for the SVM regression algorithm, we performed cross validation. The results from the SVM were reported here with 10-fold cross validation.

## Results

For prediction using support vector regressor analysis, PSG data from a total of 102 patients were included. Within these 102 patients, there were 47 patients with moderate severity (AHI between 15 and 30/h of total sleep time). Of these, 27 patients were male and 20 patients were female. The remaining 55 patients were severely affected with an AHI >30/h of total sleep time. Among these, 43 were male and 12 were female patients. Table 1 and Table 2 show further epidemiological data for the above patients separated by the two groups (Table 1 for the group with AHI >30/h and Table 2 for the group with AHI between 15 and 30/h).

**TABLE 1:** The epidemiological data of the 47 patients with AHI >30/h shows age in years, BMI in kg/m^2^, CVRF for the number of cardiovascular risk factors (hypertension, obesity, diabetes mellitus, hyperlipoproteinemia), AHI in number per hour, RDI (Respiratory Disturbance Index) in number per hour, TST (total sleep time) in minutes, ODI (oxygen desaturation index) in number per hour and Arousal Index in number per hour.

	N	Minimum	Maximum	Mean value	Standard deviation
Age (years)	47	28	70	50,17	10,85
BMI (kg/m^2)	47	20,93	46,57	30,99	5,60
CVRF (n)	47	0	3	1,23	0,87
AHI (n/h)	47	14,4	31,3	20,79	4,29
RDI (n/h)	47	15,0	32,7	21,40	4,36
TST (min)	47	202,5	491,0	362,33	54,21
ODI (n/h)	47	1,4	29,8	12,84	7,28
Arousal Index (n/h)	47	4,3	42,5	22,19	7,42

**TABLE 2:** The epidemiological data of the 55 patients with AHI between 15 and 30/h shows age in years, BMI in kg/m^2^, CVRF for the number of cardiovascular risk factors (hypertension, Obesity, diabetes mellitus, hyperlipoproteinemia), AHI in number per hour, RDI (Respiratory Disturbance Index) in number per hour, TST (total sleep time) in minutes, ODI (oxygen desaturation index) in number per hour and Arousal Index in number per hour.

	N	Minimum	Maximum	Mean value	Standard deviation
Age (years)	55	27	86	52,91	12,94
BMI (kg/m^2)	55	19,57	43,27	31,70	5,12
CVRF (n)	55	0	5	1,77	1,22
AHI (n/h)	55	16,20	130,40	51,07	21,70
RDI (n/h)	55	16,9	130,4	51,89	21,08
TST (min)	55	166,5	458,0	336,86	65,38
ODI (n/h)	55	8,6	97,5	40,44	24,27
Arousal Index (n/h)	55	9,9	93,4	38,19	18,50

**FIGURE 3:**
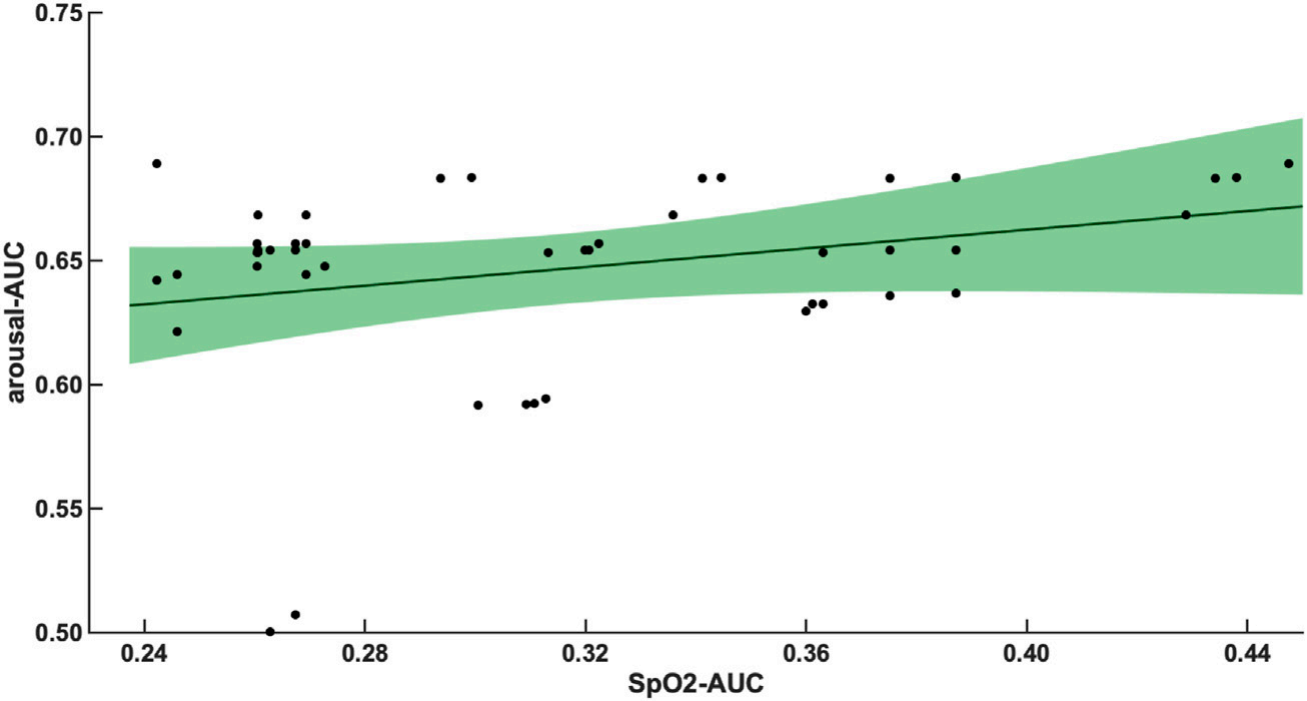
Shows the linear regression results between the arousal-AUC and SpO2-AUC for patients with AHI 15–30/h of TST. The green margins indicate the standard deviation for the correlation and the black dots indicate each subject in this group. r = 0.280/*p* = 0.056/*n* = 47.

**FIGURE 4:**
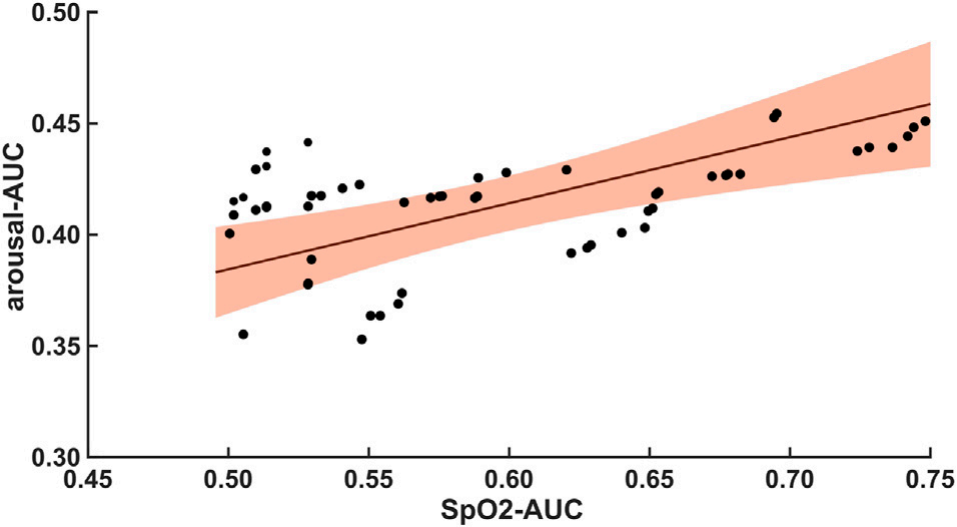
Shows the linear regression results between the arousal-AUC and SpO2-AUC for patients with AHI >30/h of TST. The orange margins indicate the standard deviation for the correlation and the black dots indicate each subject in this group. r = 0.404/*p* = 0.002/*n* = 55.

**FIGURE 5:**
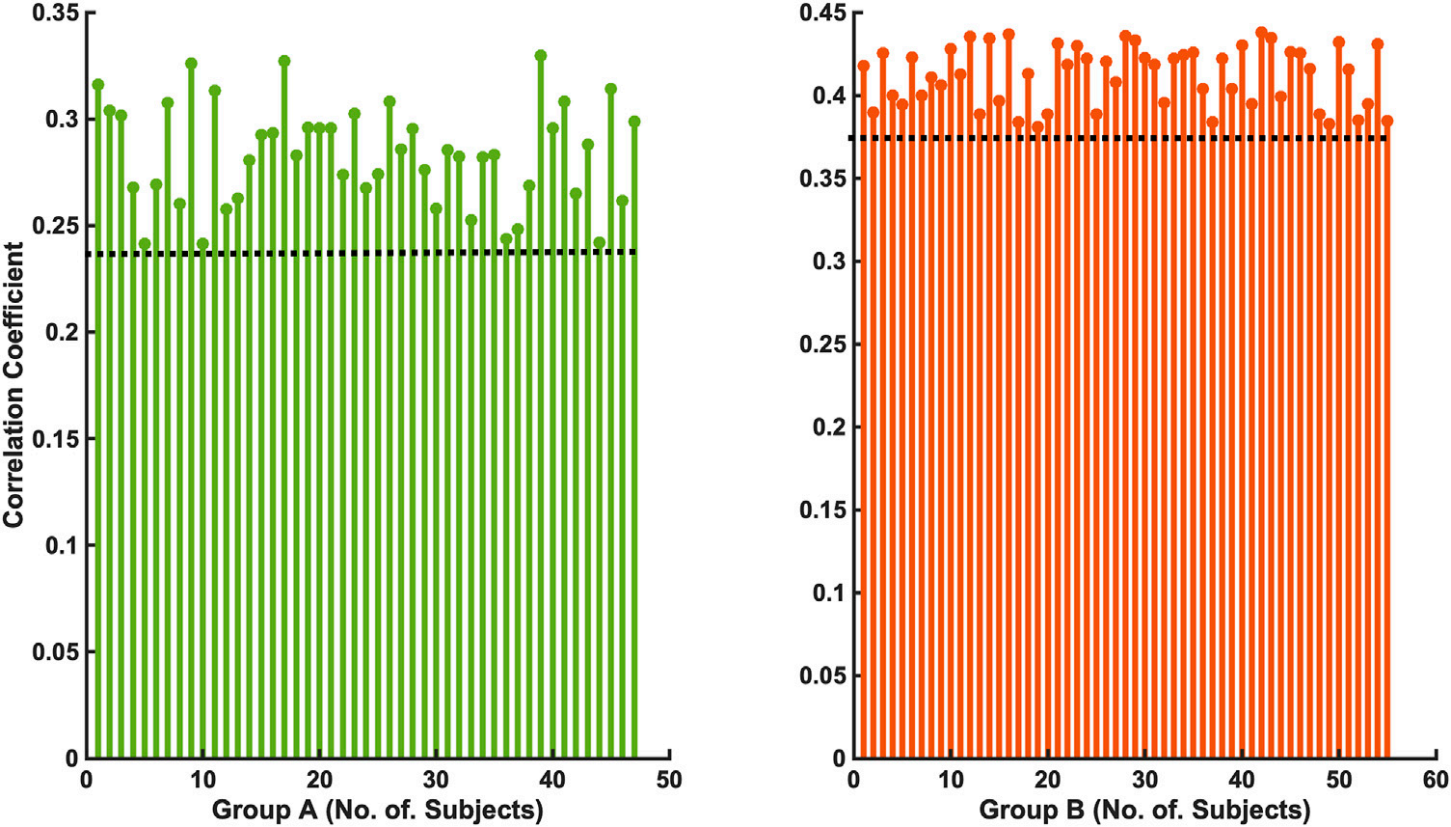
Shows the correlation coefficient between the arousal-AUC and SpO2-AUC for all arousals of every individual of the two separate groups (Group A: AHI 15–30/h of TST; Group B: AHI >30/h of TST).

The 47 patients with moderate severity of AHI between 15 and 30/h of total sleep time, showed a weak and non-significant correlation between the arousal-AUC of EEG C3/C4 pooled trace and SpO2-AUC of PSG (r = 0.280; *p* = 0.056), which is shown in Figure 3. In contrast, the remaining 55 severely affected patients, with an AHI >30/h of total sleep time, showed a significant correlation in linear regression of the arousal-AUC of EEG C3/C4 pooled trace and SpO2-AUC of PSG (r = 0.404; *p* = 0.002), shown in Figure 4. Figure 5 shows the correlation coefficient between the arousal-AUC and SpO2-AUC for all arousals of every individual of the two separate groups.

**FIGURE 6:**
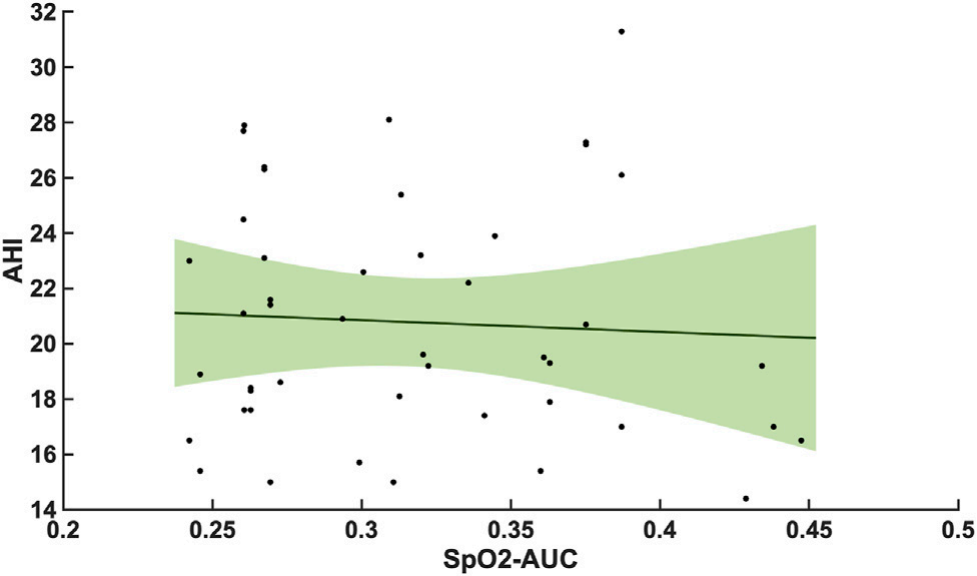
Shows the linear regression results between the AHI and SpO2-AUC for patients with AHI 15–30/h of TST. The green margins indicate the standard deviation for the correlation and the black dots indicate each subject in this group. r = -0,0580/*p* = 0,6987/*n* = 47.

**FIGURE 7:**
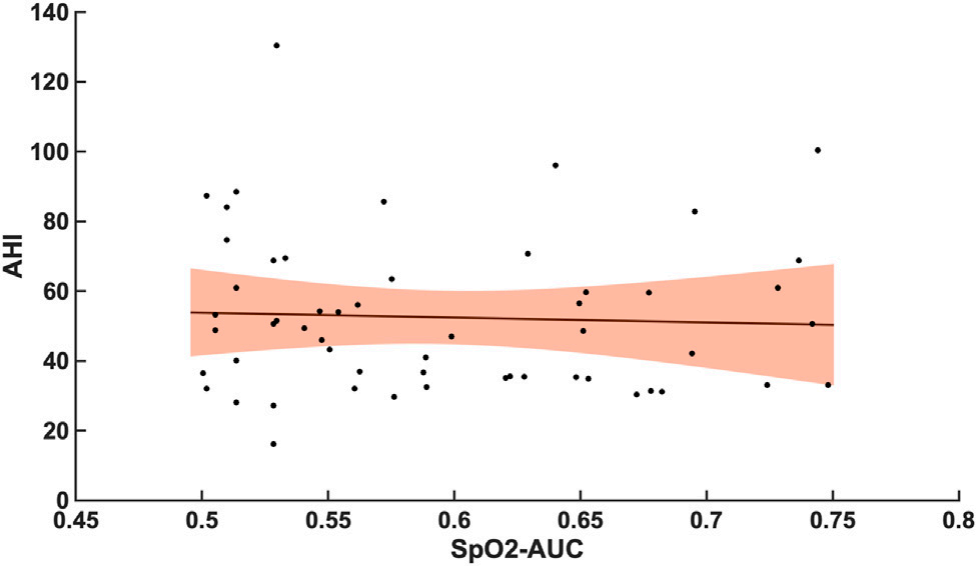
Shows the linear regression results between the AHI and SpO2-AUC for patients with AHI >30/h of TST. The orange margins indicate the standard deviation for the correlation and the black dots indicate each subject in this group. r = -0,0480/*p* = 0.728/*n* = 55.

Compared with the correlation between arousal-AUC and SpO2-AUC, the correlation between AHI and SpO2-AUC offered no to little linear correlation for both groups and did so without significance (group A [r = -0,0580; *p* = 0,6987] and group B [r = -0,0480; *p* = 0.728]). Figure 6 and Figure 7 show the linear regression results between the AHI and SpO2-AUC for both groups.

**FIGURE 8:**
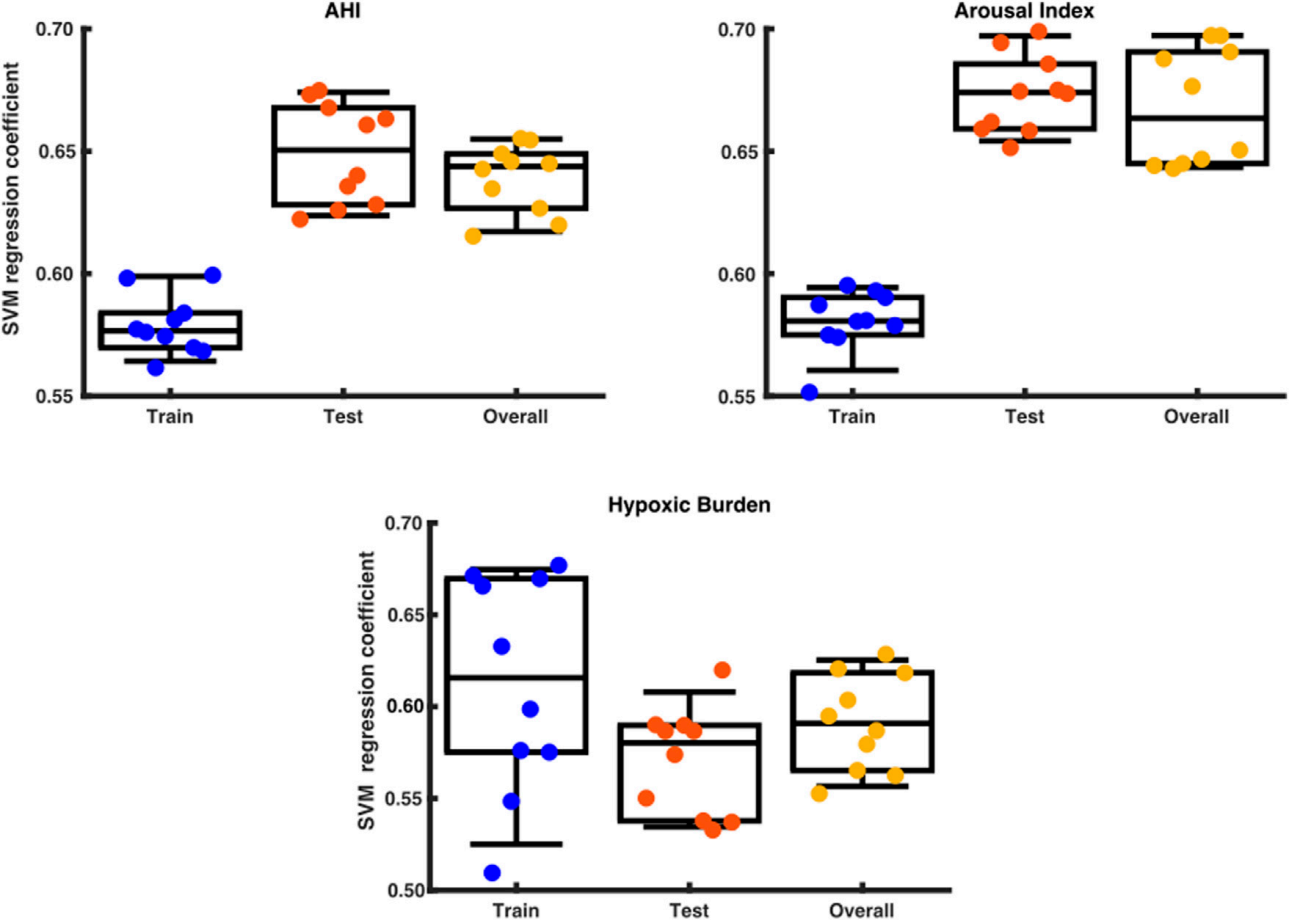
Box plot with the distribution of a data set. The x axis shows the division into training set, test set and overall testing. The y-axis shows the SVM correlation coefficient.

By using a support vector regressor (SVR) analysis with the arousal-AUC metric we could predict the AHI with an correlation coefficient of 0,58 in the training set, 0,65 in the testing set and 0,64 overall. The hypoxic burden showed a correlation coefficient of 0,62 in the training set, 0,58 in the testing set and 0,59 overall. The arousal index had a correlation coefficient to the arousal-AUC metric of 0,58 in the training set, 0,67 in the testing set and 0,66 overall. Figure 8 shows a box plot with the distribution of a data set.

## Discussion

Our results show that using an SVR with the arousal-AUC metric results in very high predictive power for the AHI, hypoxic burden, and arousal index and therefore could be used as an additional quantitative surrogate marker in the description of obstructive sleep apnea respiratory disease severity.

A significant correlation between the arousal-AUC and SpO2-AUC was found in patients with AHI >30/h than in patients with AHI between 15/h and 30/h. Therefore, especially in OSAS patients with severe respiratory distress, a novel positive correlation between the hypoxic burden, as represented by the SpO2-AUC-metric, and the severity of arousal (as represented by the arousal-AUC) was found (Figures 3, 4). In comparison, we were able to show with the data that the AHI to SpO2-AUC offered no significant correlation. This again underlines, as described in the introduction, that the AHI as a singular parameter is not suitable for the description of obstructive sleep apnea and is inferior to new parameters, which could be used complementarily. To further understand the origin of arousal, this correlation can serve as another building block to show that there is a close relationship between hypoxia and arousal. However, it cannot be used to conclude a causal relationship.

Given that hypoxic events usually temporally precede respiratory arousals in OSAS patients, it may well be argued that the greater the hypoxic burden, the greater becomes the central nervous system (CNS) drive trying to compensate the hypoxic burden by means of an arousal. To our knowledge, there are no previous reports on such a correlation. There is a much lesser degree of this precise correlation in OSAS patients with moderate (AHI = 15–30/h) respiratory distress. This suggests that, in OSAS patients with moderate respiratory disease severity the degree and/or the temporal extent of oxygen desaturations may exert a much less significant stimulatory influence on the CNS arousal-generating drive than in patients with severe OSAS. As a result, either different pathophysiologic mechanisms or dose-effect responses regarding regulation of arousal features by hypoxia in OSAS may exist in patients with different degrees of respiratory OSAS severity.

Younes et al. showed that the average arousal intensity is not related to the magnitude of the preceding respiratory stimuli but was positively associated with arousal duration, time to arousal, rate of change in epiglottic pressure, and negatively with body mass index (R2 > 0.10, *p* ≤ 0.006). The authors concluded that the average arousal intensity is independent of the preceding respiratory stimulus. (Younes, 2004). The same could be observed in other studies (Amatoury et al., 2016). It is also noted that respiratory-induced cortical arousals occur during inspiration as well as expiration but differ in the increase of the tensor palatini muscle activity and minute ventilation (Amatoury et al., 2018). With the knowledge from recent studies that arousal has a strong correlation with sympathetic hyperactivity in OSA patients and thus a possible component in pathophysiological cause, it can be assumed that also here, similar to the AHI, not only the total number of events, but the microstructure of the event plays a significant role in the disease process (Kim et al., 2019; Ferreira et al., 2020). Given the good correlation coefficient between AUC-arousal and the AHI, hypoxic burden, and the arousal index, it may be assumed that AUC-arousal is related to the severity of OSA. These facts support the hypothesis that not only the intensity alone or duration alone of an arousal, but the whole microstructure is relevant to understand the possible arousal “burden”.

One strength of the study is that we used a pooled C3- and C4- EEG signal for the analysis of the data, which resulted in optimal use of the available information provided by any one of the two brain hemispheres (Prucnal and Polak, 2019). The data originated from a rather large group of 102 patients. A very homogeneous distribution of the measured values in terms of AHI, age, and BMI resulted. A weakness of this study is that no mildly affected patients with an AHI <15/h were studied; nonetheless, we have made the conscious decision not to study such a group of patients from the very beginning because we knew from previous research and our own experience that such patients have much less frequent arousals, making statistical analysis of arousals in such a mild OSA patient subgroup quite difficult or impossible. Additionally, a larger patient population would of course further enhance the correlation coefficient, as well as the results of the SVM.

Support vector machine algorithms have been increasingly applied in medical data during the past few years since they can provide systematized architecture for analyzing and extracting important information from complex data (Divya and Sonali, 2013; Huang et al., 2020). For this reason, a support vector regressor analysis was deliberately chosen in this study, on the one hand with the intention of making the best possible prediction, and on the other hand to demonstrate the possibilities provided by machine learning methods. In the future, this could also improve and simplify the previous, visually manual analysis of polysomnography.

A larger cohort would be needed to improve the power of the study. Future studies should further dissect the microstructure of arousal and compare it with symptomatic components of OSA. Given the myriad of OSA-associated conditions across multiple biological systems, one might expect the optimal metric of OSA severity to differ depending on the outcome of interest (Malhotra et al., 2021).

## Conclusion

Given that traditional metrics, such as the AHI or oxygen desaturation index (ODI), increasingly appear to be insufficient to capture the complexity of the OSAS disorder in many patients, the arousal-AUC metric may provide a novel additional strong correlate for the hypoxic burden that should be validated in further studies.

## Data Availability

The raw data supporting the conclusions of this article will be made available by the authors, without undue reservation.
